# Avascular necrosis of the talus causing meniscoid lesions in the ankle joint: a case report

**DOI:** 10.1186/s13256-022-03298-7

**Published:** 2022-02-26

**Authors:** Ceyran Hamoudi, Andrei Doljencu, Tamás Illes

**Affiliations:** Department of Orthopaedics, Brugmann University Hospital, Brussels, Belgium

**Keywords:** Avascular necrosis, Talus, Ankle injury, Meniscoid lesion, Case report

## Abstract

**Background:**

Meniscoid lesions have been reported in patients with chronic ankle injuries, especially in soccer athletes, and such lesions cause soft-tissue impingement and pain. To our knowledge, we are the first to report a meniscoid lesion in the ankle joint presenting as a long-term sequela of avascular necrosis of the talus that developed in childhood.

**Case presentation:**

In this paper, we describe a 55-year-old Caucasian male patient who presented with a 1-year history of intermittent locking, “giving way,” weight-bearing pain, and swelling over the anterior aspect of the ankle joint. Imaging showed a rare case of avascular necrosis of the talus associated with an unstable plica-like lesion that was removed arthroscopically after unsuccessful conservative treatment.

**Conclusion:**

We demonstrate that unstable meniscoid lesions of the ankle joint can be treated successfully with arthroscopic debridement. We obtained satisfactory short-term clinical results at the 2-year follow-up, even though advanced osteoarthritis was present.

## Background

Avascular necrosis (AVN) of the talus is a challenging condition to treat, and is usually associated with major disabilities in patients. Fracture of the talus is the most common cause of AVN, accounting for up to 75% of cases [1]. Anatomically, the talus is predisposed to ischemia due to its particular blood supply [2]. Complications of AVN can lead to serious disabilities, especially when associated with talar dome collapse, ankle degenerative changes, arthritis, instability, and pain [3]. Meniscoid lesions have been reported in chronic ankle injuries, especially in soccer athletes, causing soft-tissue impingement and pain [4]. In this case report, we present a rare case of a meniscoid lesion in the ankle presenting as a long-term sequela of traumatic AVN of the talus, and satisfactory recovery of the patient following arthroscopic treatment.

## Case presentation

### Patient information, clinical findings, timeline, and diagnostic assessment

A 55-year-old Caucasian male presented for consultation with a 1-year history of intermittent locking, “giving away,” weight-bearing pain, and swelling over the anterior aspect of his right ankle that had recently been aggravated. The patient was a sedentary worker and non-athlete. He stated that he had experienced right ankle trauma at 8 years old, following an accidental fall from a height that was never investigated or treated. His pain and swelling had disappeared after several months, and the patient never had any further symptoms until recently.

Physical examination revealed pain over the anterior aspect of the ankle joint. No snap was audible, and no lateral instability was exhibited. Provocative tests of the flexor hallucis longus were painless, dorsiflexion was slightly limited and painful, and forced plantar flexion was full and painless. Moreover, we noted pain with a single leg squat. There was no proprioception impairment, postural control difficulty, or strength deficit. Shortening of the right inferior limb was measured to be approximately 1 cm.

**Fig. 1:**
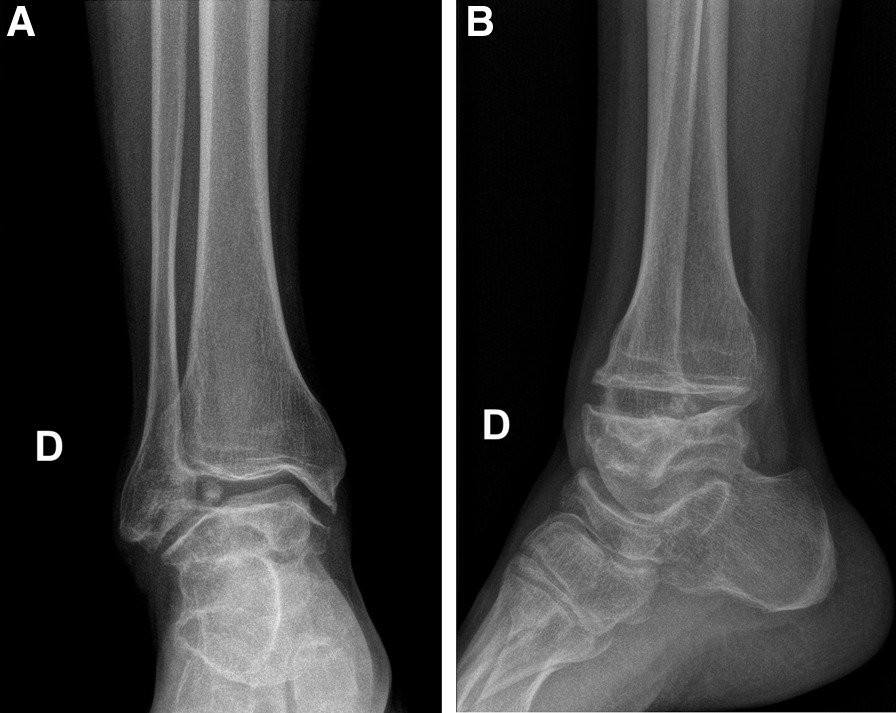
**A** Plain AP view of the ankle joint. **B** Plain lateral view of the ankle joint. **D** Right side (in French: Droit)

**Fig. 2:**
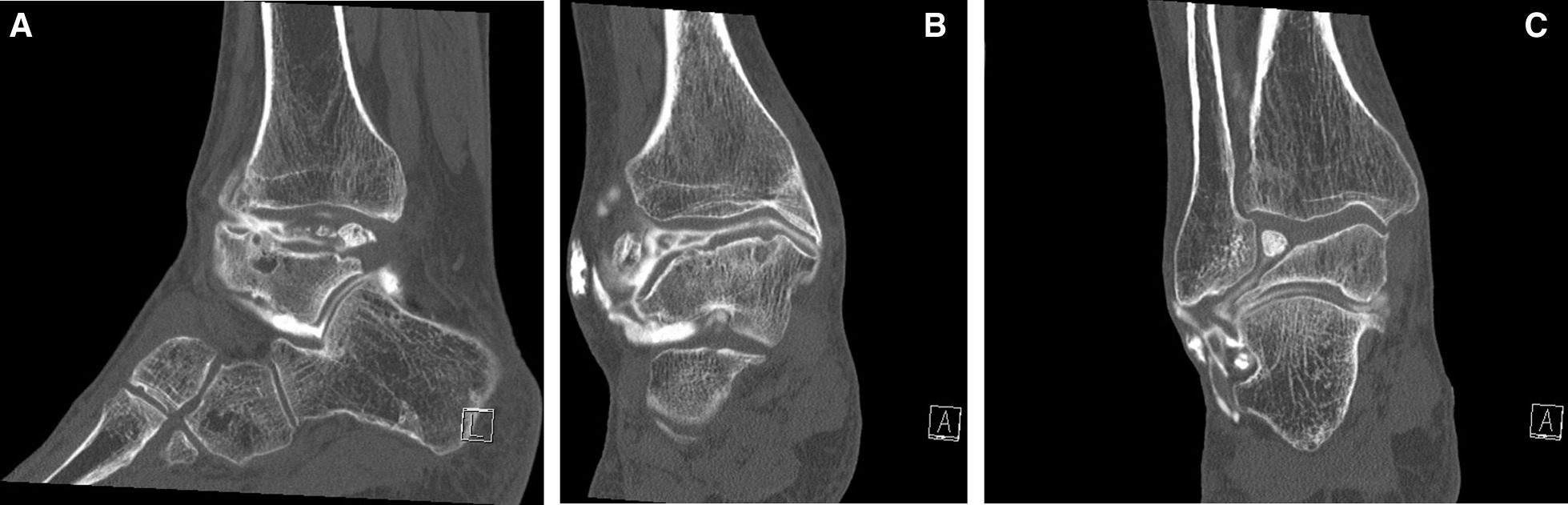
**A** Computed tomographic arthrography (sagittal image) of the ankle shows collapse of the talar dome, subchondral cysts, anterior fibrous-like tissues, and numerous posterior accessory ossicles. **B** Computed tomographic arthrography (frontal image) of the ankle shows subchondral talar cysts, meniscoid lesions, and diffuse intraarticular fibrosis. **C** Computed tomographic arthrography (frontal image) of the ankle shows an anterior intraarticular ossicle.

Plain radiographs (Fig. 1a and b) showed marked flattening of the posterior part of the talus associated with osteoarthritis of the tibiotalar joint with osteophytes and subchondral sclerosis. More importantly, we visualized an obvious 1.5 cm intraarticular ossicle. The posterior calcaneal tuberosity was prominent, but the patient did not have related clinical symptoms. Computed tomographic arthrography of the ankle (Fig. 2a–c) revealed collapse of the talar dome with an irregular surface and advanced osteoarthritis associated with subchondral cysts. The images also showed anterior ankle impingement, numerous posterior accessory ossicles, and lesions of the anterior tibiofibular, anterior talofibular, and calcaneofibular ligaments. Moreover, they revealed slight dysplasia of the navicular bone and diffuse intraarticular fibrosis.

### Therapeutic intervention

**Fig. 3:**
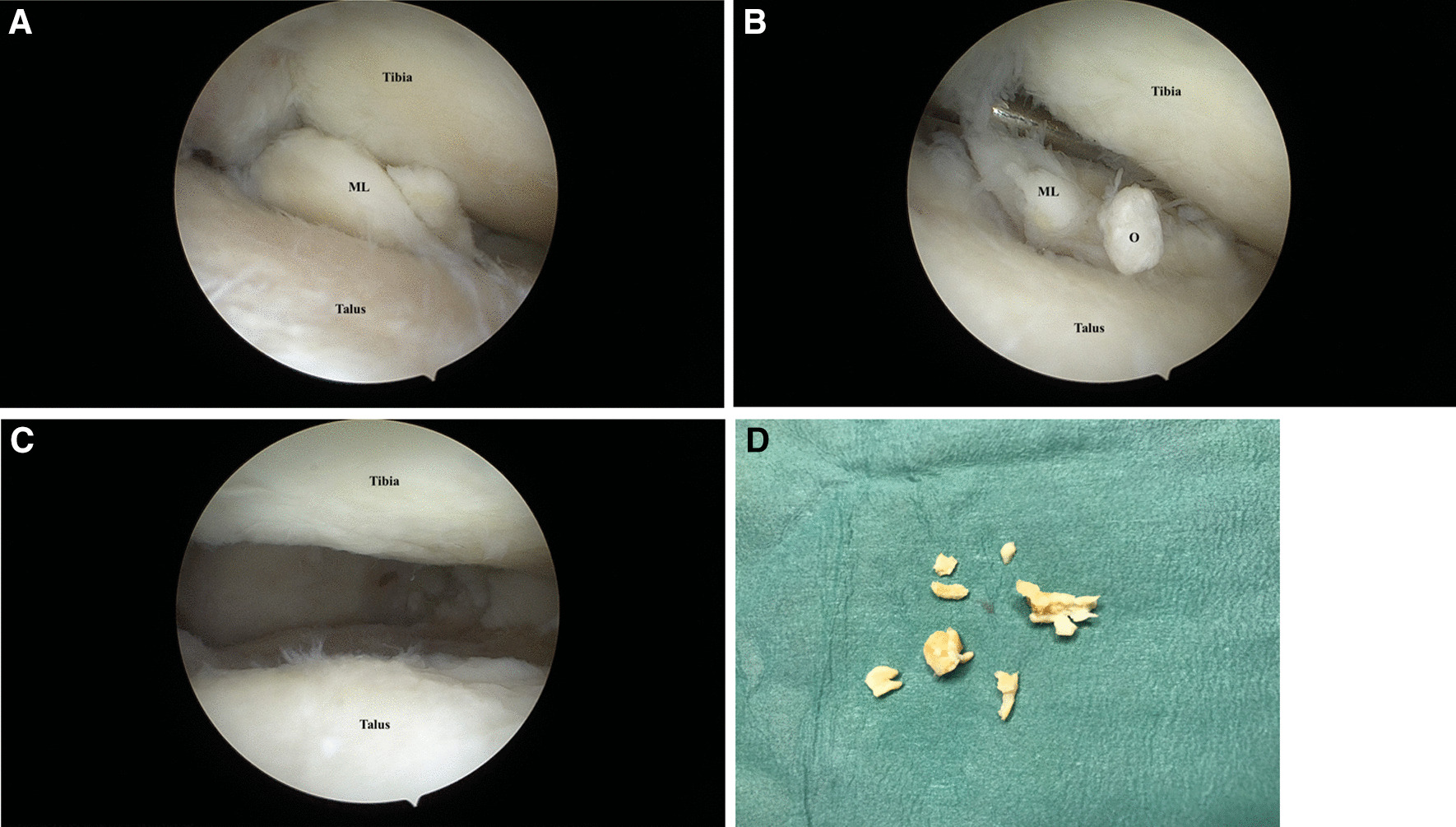
**A** View of the ankle joint during arthroscopy before debridement. ML indicates the meniscoid lesion. **B** View of the ankle joint during arthroscopy before debridement. *ML* meniscoid lesion, *O* ossicle. **C** View of the ankle joint during arthroscopy after debridement. **D** Excised meniscus-like tissue from the ankle joint

After 6 months of conservative treatment, including rehabilitation, pain medication, and intraarticular corticoid injection, the patient still had the same complaints. Surgical arthroscopy was chosen for debridement. An ambulatory arthroscopic intervention was performed under general anesthesia. A tourniquet was applied for hemostasis. Two portals were used: anterolateral and anteromedial. At the time of surgery, no instability was seen during anesthesia. Upon arthroscopic examination through the anterior medial portal, an unstable band of white, meniscus-like tissue accompanied by an unstable 1.5 cm ossicle (Fig. 3a–d) was found between the fibula and the talus, which was removed through the arthroscope via the anterior lateral portal. In this context, the ankle anatomy was abnormal, making it difficult to distinguish cartilage from fibrous tissue. Synovitis was present, and we noted stage II–III chondromalacia throughout the ankle joint. The patient was discharged the same day with pain medication and full weight bearing. Immediate postoperative rehabilitation was implemented.

### Follow-up and outcomes

**Fig. 4:**
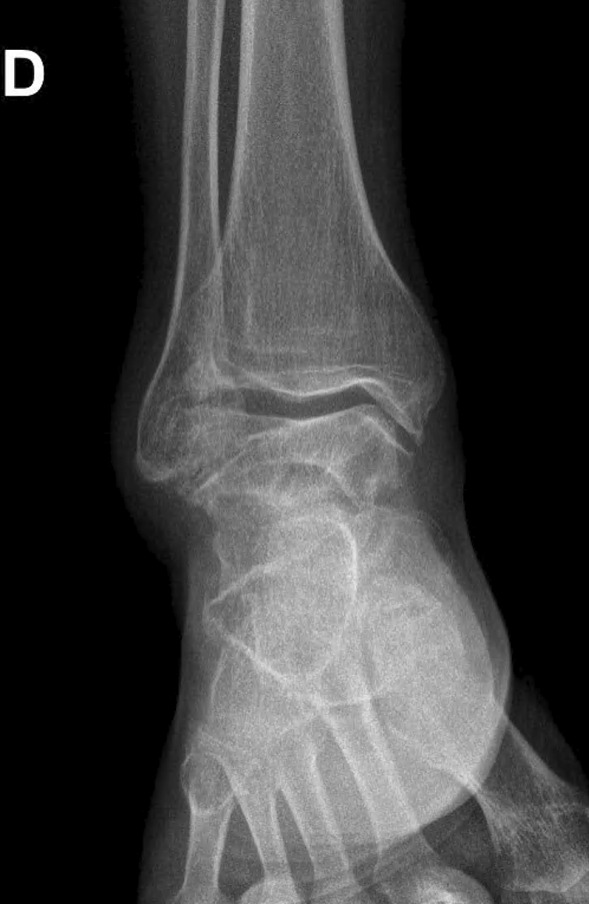
**A** Plain AP view of the ankle joint after debridement. **D** Right side (in French: Droit)

The follow-up radiograph (Fig. 4a) and clinical follow-up at 1 and 2 years were satisfactory. The patient did not complain of ankle instability, and he reported experiencing pain and swelling only on rare occasions.

## Discussion and conclusions

Meniscoid lesions in the ankle were first reported in the literature by Wolin *et al*. in 1950 [5]. Since then, most reported cases have been in professional soccer players as a result of repetitive extreme ankle dorsiflexion [4]. Other sports that have been outlined are dance and gymnastics, in which athletes do not use footwear [6]. Acute injuries have been shown to lead to residual ankle weakness and instability with a higher risk of recurrent injuries, turning acute injuries into more chronic injuries. Meniscoid lesions formed in the anterior ankle can potentially cause soft tissue impingement syndrome. This is caused by soft tissue hypertrophy and secondary fibrosis of tissue, which then becomes trapped between the talus and malleolus [7]. We have not found any reports in the literature of meniscoid lesions in the ankle joint as a long-term sequela of avascular necrosis of the talus. In 1991, Gächter and Gerber [8] identified a condition they called plica syndrome, which is a meniscoid lesion developing following a posttraumatic event. In most cases, concomitant chronic synovitis is not resolved by conservative treatment [7]. McCarrol *et al*. [4] advocate arthroscopy as a means of removing meniscus-like tissue found in the ankle, followed by a short rehabilitation program. Studies show successful results after a 2-year follow-up period. In our case, we followed a similar protocol, although our patient presented concomitant degenerative changes. In our patient, the talar AVN caused a collapse of the talar dome, leading to degenerative changes in both the ankle and subtalar joints, as well as a shortening of the affected limb. AVN of the talus in children is rare. Schmidt *et al*. [9] reported that the incidence varies between 0.01% and 0.08%. Our patient was 8 years old when he sustained accidental ankle trauma caused by a fall from a height. Given his age, the talus was not fully developed, and we know from the research of Rammelt *et al*. [10] that children are more prone to AVN than adults. Treatment of talar AVN is complex, especially when combined with a collapse of the talar dome and body and with subsequent subtalar and ankle osteoarthritis, as in our case. Tibiotalar fusion is considered the gold standard treatment for late-stage ankle arthritis in young and active patients [11] and has shown reliable results in osteonecrosis [1]. According to Dhillon *et al*. [12], in the post-collapse stage, some salvage options such as subtalar, Blair’s tibiotalar, tibiocalcaneal, tibiotalocalcaneal, or Blair’s fusion, are viable options. Furthermore, talar replacement is another possible option with promising results [13, 14], whereas standard total ankle arthroplasty is usually contraindicated in talar AVNs [11].

In this case report, we present a case of an unstable preexisting meniscoid lesion accompanied by an unstable ossicle as the long-term sequela of AVN of the talus. This case initially developed in childhood and became more symptomatic in adulthood. Both lesions led to fibrous and osseous impingements that were removed arthroscopically. The treatment was a success, with the patient complaining of only very rare pain and swelling at the 2-year follow-up. This result supports the role of arthroscopy in symptomatic meniscoid lesions in the ankle joint as a first line of surgical treatment. However, for later end-stage ankle arthritis, more invasive measures such as tibiotalar fusion may be needed.

## Data Availability

Data sharing was not applicable to this article, as no datasets were generated or analysed during the current study.

## Abbreviation

AVN: Avascular necrosis
